# Antiadhesive activity of the biosurfactant pseudofactin II secreted by the Arctic bacterium *Pseudomonas fluorescens *BD5

**DOI:** 10.1186/1471-2180-12-24

**Published:** 2012-02-23

**Authors:** Tomasz Janek, Marcin Łukaszewicz, Anna Krasowska

**Affiliations:** 1 Faculty of Biotechnology, University of Wroclaw, Przybyszewskiego 63/77, Wroclaw 51-148, Poland; 2 Faculty of Chemistry, Wroclaw University of Technology, Gdańska 9/7, Wroclaw 50-344, Poland

**Keywords:** Biosurfactant, Lipopeptide, Adhesion, Biofilm, Uropathogenic microorganisms

## Abstract

**Background:**

Pseudofactin II is a recently identified biosurfactant secreted by *Pseudomonas fluorescens *BD5, the strain obtained from freshwater from the Arctic Archipelago of Svalbard. Pseudofactin II is a novel compound identified as cyclic lipopeptide with a palmitic acid connected to the terminal amino group of eighth amino acid in peptide moiety. The C-terminal carboxylic group of the last amino acid forms a lactone with the hydroxyl of Thr3.

Adhesion is the first stage of biofilm formation and the best moment for the action of antiadhesive and anti-biofilm compounds. Adsorption of biosurfactants to a surface e.g. glass, polystyrene, silicone modifies its hydrophobicity, interfering with the microbial adhesion and desorption processes. In this study the role and applications of pseudofactin II as a antiadhesive compound has been investigated from medicinal and therapeutic perspectives.

**Results:**

Pseudofactin II lowered the adhesion to three types of surfaces (glass, polystyrene and silicone) of bacterial strains of five species: *Escherichia coli, Enterococcus faecalis, Enterococcus hirae, Staphylococcus epidermidis, Proteus mirabilis *and two *Candida albicans *strains. Pretreatment of a polystyrene surface with 0.5 mg/ml pseudofactin II inhibited bacterial adhesion by 36-90% and that of *C. albicans *by 92-99%. The same concentration of pseudofactin II dislodged 26-70% of preexisting biofilms grown on previously untreated surfaces. Pseudofactin II also caused a marked inhibition of the initial adhesion of *E. faecalis, E. coli, E. hirae *and *C. albicans *strains to silicone urethral catheters. The highest concentration tested (0.5 mg/ml) caused a total growth inhibition of *S. epidermidis*, partial (18-37%) inhibition of other bacteria and 8-9% inhibition of *C. albicans *growth.

**Conclusion:**

Pseudofactin II showed antiadhesive activity against several pathogenic microorganisms which are potential biofilm formers on catheters, implants and internal prostheses. Up to 99% prevention could be achieved by 0.5 mg/ml pseudofactin II. In addition, pseudofactin II dispersed preformed biofilms. Pseudofactin II can be used as a disinfectant or surface coating agent against microbial colonization of different surfaces, e.g. implants or urethral catheters.

## Background

Biofilms, which are formed by the majority of microorganisms in natural environments, are structures with low sensitivity to drugs [1]. Many laboratories are synthesizing or isolating new compounds preventing the formation of biofilms or causing their elimination [2,3]. Adhesion is the first stage of biofilm formation and the best moment for the action of antiadhesive and anti-biofilm compounds. Biosurfactants are promising compounds often showing antimicrobial and antiadhesive properties and sometimes penetrating and removing mature biofilms [4]. Microbial surfactants-amphiphilic, surface-active, secondary metabolites of bacteria or fungi ranging from low-molecular-mass glycolipids, sophorolipids, rhamnolipids and lipopeptides, to high-molecular-mass proteins, lipopolysaccharides and lipoproteins [5]-can interact with interfaces and inhibit the adhesion of microorganisms to different surfaces. They are an alternative to synthetic surface-active agents because of their low toxicity and biodegradability [6].

Another mechanism of biosurfactant action is the permeabilization of bacterial cells. The rhamnolipid secreted by *Pseudomonas *sp. S-17 permeabilized Gram-negative and Gram-positive cells, but a strong inhibition of growth was observed only in the case of Gram-positive bacteria [7]. Biofilm disruption was observed after the addition of rhamnolipids from *Pseudomonas aeruginosa *[8] and lipopeptide from *Bacillus *spp. [9]. A particular group of biosurfactants, lipopeptides, can act as antibiotics and also as antiviral [10] and antitumor agents [11]. Surfactin from *Bacillus subtilis *can interact with the plasma membranes of bacterial and fungal cells leading to their disruption [12]. The effects of biosurfactants on decreased microbial adhesion and detachment from different surfaces can be conveniently utilized in many fields, from medicine to various branches of industry, e.g., antimicrobial or antitumor activities [13,14] and their surface activity and antiadhesive properties can be suitable for preventing microbial colonization of implants or urethral catheters. Microbial surfactants from *Lactobacillus fermentum *and *Lactobacillus acidophilus *adsorbed on glass, reduced the number of adhering uropathogenic cells of *Enterococcus faecalis *by 77% [15]. A surfactant released by *Streptococcus thermophilus *has been used for fouling control of heat-exchanger plates in pasteurizers as it retards the colonization of other thermophilic strains of *Streptococcus *responsible for fouling [16].

Within the collection of 132 morphologically distinct bacteria isolated from water and ground provided from the wild environment around the University of Wroclaw scientific base in the Arctic Archipelago of Svalbard [17], *Pseudomonas fluorescens *BD5 was identified as strain which strongly reduces surface tension of its culture supernatant [18]. We purified and identified the chemical structure of two new *P. fluorescens *BD5 biosurfactants, pseudofactin I and II [19]. Both compounds are cyclic lipopeptides with a palmitic acid connected to the terminal amino group of an octapeptide. The C-terminal carboxylic group of the last amino acid (Val or Leu) forms a lactone with the hydroxyl of Thr3. The biosurfactant was found to be stable within the range from -20°C to 100°C, had the minimum surface tension (31.5 mN/m) and the critical micelle concentration (72 mg/L) [19]. Emulsification activity and stability of pseudofactin II was greater than that of the synthetic surfactants such as Tween 20 and Triton X-100.

The aim of this paper was to assess how the pseudofactin II influences the adhesion and biofilm formation of microorganisms such as *Escherichia coli, E. faecalis, Enterococcus hirae, Staphylococcus epidermidis, Proteus mirabilis, Vibrio ordalii, Vibrio harveyi *and *Candida albicans *found in gastrointestinal and urinary tract. Since the effects of a surfactant may differ depending on both the type of the microorganism and the type of surface it adheres to, we tested its action on the adherence of the above pathogenic microorganisms to three types of surfaces, polystyrene, glass (as standard laboratory surfaces for adhesion tests) and silicone (used in medical application such as urethral catheters).

## Methods

### Microorganisms and culture conditions

*P. fluorescen*s BD5 strain was obtained from freshwater from the Arctic Archipelago of Svalbard [19] and maintained on the mineral salts medium MSM (7 g/L K_2_HPO_4_, 2 g/L KH_2_PO_4_, 1 g/L (NH_4_)_2_SO_4_, 0.5 g/L sodium citrate 2H_2_O, and 0.1 g/L MgSO_4_.7H_2_O) with 2% D-glucose.

The antimicrobial and antiadhesive properties of pseudofactin II were tested on several pathogenic strains that colonize animals gastrointestinal tract or medical devices. *E. coli *ATCC 25922, *E. coli *ATCC 10536, *E. coli *17*-*2 (clinical isolate, Wroclaw Medical University), *E. faecalis *ATCC 29212, *E. faecalis *JA/3 (clinical isolate, Wroclaw Medical University), *E. hirae *ATCC 10541, *S. epidermidis *KCTC 1917 [20], *P. mirabilis *ATCC 21100 were grown at 37°C and *V. harveyi *ATCC 14126*, V. ordalii *KCCM 41669 were grown at 28°C in LB medium (10 g/L bacto-tryptone, 5 g/L bacto-yeast extract, 10 g/L NaCl). Two fungal strains, *C. albicans *ATCC 20231 and *C. albicans *SC5314 [21], were grown in a 6.7 g/L yeast nitrogen base (YNB, pH 5.5), broth (Difco Laboratories) containing 2% D-glucose for adhesion tests. To prevent filamentation of *C. albicans*, pre-culture was incubated at 28°C, while experiments with biofilms were performed at 37°C. RPMI-1640 medium (Cambrex, Verviers, Belgium) was used for *Candida *biofilms formation.

### Isolation and purification of pseudofactin II

Pseudofactin II produced by *P. fluorescens *BD5 was obtained by extraction of cell free supernatant by ethyl acetate and evaporation of the extract under vacuum. The crude biosurfactant was separated by RP-HPLC in the same manner as reported earlier [19]. Purified pseudofactin II fraction was dried and stored at -20°C for further studies. Analytical RP-HPLC (data not shown) of purified pseudofactin II showed that its purity was > 99%.

### Antimicrobial assays

The antimicrobial activity of isolated pseudofactin II was determined by the microdilution method in 96-well flat-bottomed plastic microplates (Sarstedt, Nümbrecht, Germany). Briefly, 50 μl volumes of sterile double strength LB (for bacterial) or YNB (for yeast) medium were dispensed into the wells of a 96-well microplate. Subsequently, 50 μl volumes of pseudofactin II (0.035 to 0.5 mg/ml) solution in phosphate-buffered saline (PBS) were added to the microplate wells and mixed with the medium. Negative and growth control wells did not contain biosurfactant. All wells (except for negative controls) were inoculated with 2 μl of overnight bacterial or yeast cultures (diluted to OD_600 _= 0.1) in LB or YNB medium respectively, and the microplates were incubated for 24 h at 37°C or 28°C for bacterial or yeast cultures, respectively. After 24 h of incubation, the optical density at 600 nm of each well was measured using an Asys UVM 340 (Biogenet) microplate reader. The growth inhibition percentages at different pseudofactin II concentrations for each microorganism were calculated as:

$$\%\;\text{growth}\;\text{inhibition}=\left[1-\left(\frac{{\text{OD}}_{\text{T}}}{{\text{OD}}_{\text{C}}}\right)\right]\times 100$$

where OD_T _represents the optical density of the well with a given pseudofactin II concentration and OD_C _is the optical density of the control well (growth without pseudofactin II). Assays were carried out three times in three replicates.

### Preadhesion treatment with pseudofactin II

Inhibition of microbial adhesion by pseudofactin II was tested in 96-well plates (Sarstedt, Nümbrecht, Germany). Briefly, the wells of a sterile 96-well flat-bottom plate were filled with 100 μl of 0.035-0.5 mg/ml pseudofactin II dissolved in PBS. The plates were incubated for 2 h at 37°C on a rotary shaker (MixMate, Eppendorf, Hamburg, Germany) at 300 rpm and subsequently washed twice with PBS. Negative control (blank) wells contained pseudofactin II at the highest concentration tested (0.5 mg/ml) while positive control wells contained PBS buffer only. The overnight cultures of microbial strains were centrifuged, washed twice with PBS (pH = 7.4) and re-suspended in PBS to an optical density OD_600 _= 1.0 for bacterial and OD_600 _= 0.6 for *Candida *strains. The highest adhesion without pseudofactin II were observed at these optical densities (data not shown). A 100 μl aliquot of a washed microbial suspension was added and incubated in the wells. After a 2 h incubation at 37°C in a rotary shaker (MixMate, Eppendorf, Hamburg, Germany) at 300 rpm nonadherent cells were removed by three washes with PBS. Then the plates were stained with 0.1% crystal-violet for 5 min and again washed three times with PBS. The adherent microorganisms were permeabilized and the dye was resolubilized with 150 μl of isopropanol-0.04 N HCl and 50 μl of 0.25% SDS per well. Crystal violet optical density readings of each well were taken at 590 nm on the Asys UVM 340 (Biogenet) microplate. Pseudofactin II did not affect the absorption of negative control (crystal violet in blank wells). The microbial adhesion inhibition was calculated as growth inhibition. Assays were carried out three times in three replicates.

### Postadhesion treatment with pseudofactin II

The 96-well flat-bottomed plates were incubated for 2 h on a rotary shaker (MixMate, Eppendorf, Hamburg, Germany) at 300 rpm with 100 μl of bacterial suspension (OD_600 _= 1.0) and *Candida *suspension (OD_600 _= 0.6) in PBS at 37°C. Unattached microbial cells were removed by washing the wells three times with PBS. Next, 100 μl of 0.035-0.5 mg/ml pseudofactin II was added to each well and incubated at 37°C for 2 h on a rotary shaker (MixMate, Eppendorf, Hamburg, Germany) at 300 rpm. Control wells contained only PBS. The plates were washed three times, adherent cells were fixed with 100 μl of 0.1% crystal violet for 5 min and again washed three times with PBS. The adherent microorganisms were permeabilized and the dye was resolubilized with 150 μl of isopropanol-0.04 N HCl and 50 μl of 0.25% SDS per well. The crystal violet optical density of each well was measured at 590 nm using the microplate reader. Assays were carried out three times in three replicates. The microbial adhesion dislodging percentages at different pseudofactin II concentrations for each microorganism were calculated as:

$$\%\;\text{growth}\;\text{inhibition}=\left[1-\left(\frac{{\text{OD}}_{\text{T}}}{{\text{OD}}_{\text{C}}}\right)\right]\times 100$$

where OD_T _represents the optical density of the well with a given pseudofactin II concentration and OD_C _the optical density of the control well (without pseudofactin II). Assays were carried out three times in three replicates.

### Confocal laser scanning microscopy

**Figure 1:**
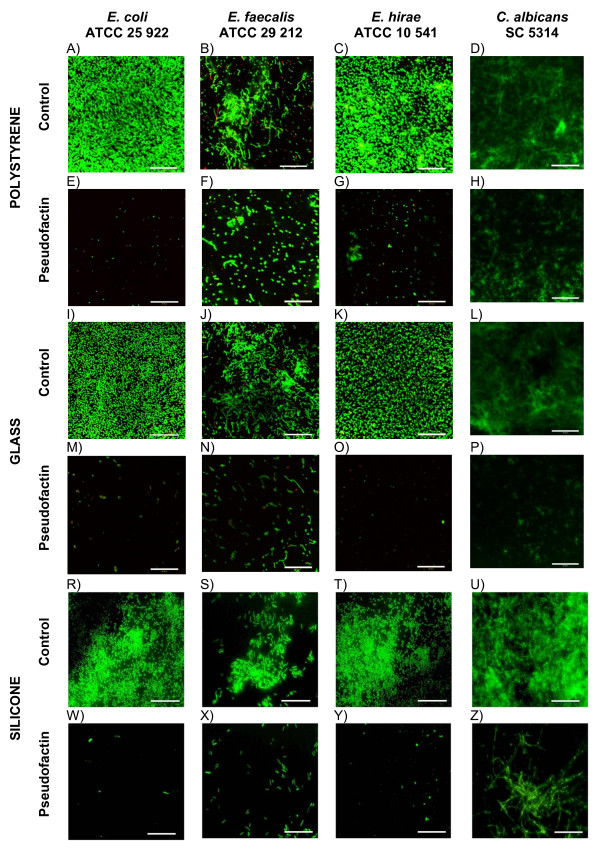
**Confocal scanning laser microscopy images of biofilm formation on polystyrene, glass microscopic coverslips and cut fragment of silicone urethral catheters by different bacterial strains: ((A, I, R) *Escherichia coli *ATCC 25922, (B, J, S) *Enterococcus faecalis *ATCC 29212, (C, K, T) *Enterococcus hirae *ATCC 10541, (D, L, U) *Candida albicans *SC5314) and biofilm inhibition after incubation with pseudofactin II (0.25 mg/ml) in the culture medium: (E, M, W) *Escherichia coli *ATCC 25922, (F, N, X) *Enterococcus faecalis *ATCC 29212, (G, O, Y) *Enterococcus hirae *ATCC 10541, (H, P, Z) *Candida albicans *mμSC5314)**. Scale bars: 50 μl.

Confocal laser scanning microscopy (CLSM) was used for visualizing the formation of bacterial and *Candida *biofilms in the absence or presence of pseudofactin II (final concentration 0.25 mg/ml) in the culture medium. Bacterial and yeast biofilms were formed on Thermanox plastic coverslips (Nalgen Nunc International Co., Rochester, NY), glass microscopic coverslips (Menzel-Glaser, Germany) and segments of silicone urethral catheters (Unomedical, Denmark) placed in wells of 24-well plates (Nalgen Nunc International Co., Rochester, NY) containing LB medium for bacteria and RPMI-1640 medium for yeast. Inocula were prepared as follows: 24 h old overnight cultures were harvested and re-suspended at normalized dilutions (OD_600 _= 0.01). Five hundred microliters inocula were injected into the wells with the coverslips and incubated for 24 h at 37°C. After this time, the coverslips were washed with PBS for 15 min. Then, the bacterial biofilms were stained for 30 min at 37°C with 1 ml of 0.6% Live/Dead BacLight viability stain (Molecular Probes, Eugene, OR) dissolved in PBS, and PBS-containing concanavalin A-Alexa Fluor 488 (Molecular Probes, Eugene, OR) conjugate (0.025 mg/ml) for *Candida *biofilms. The stained biofilms were visualized by CLSM with an Olympus FluoView 500 (Olympus Optical Co. Ltd., Japan) microscope. The CLSM used an argon ion laser at 480-490 nm for excitation and a 500-635 nm band pass filter for emission. CLSM images were processed by Olympus FluoView 500 software. Assays were carried out two times. Representative images are presented on Figure 1.

### Biofilm formation in urethral catheters

**Figure 2:**
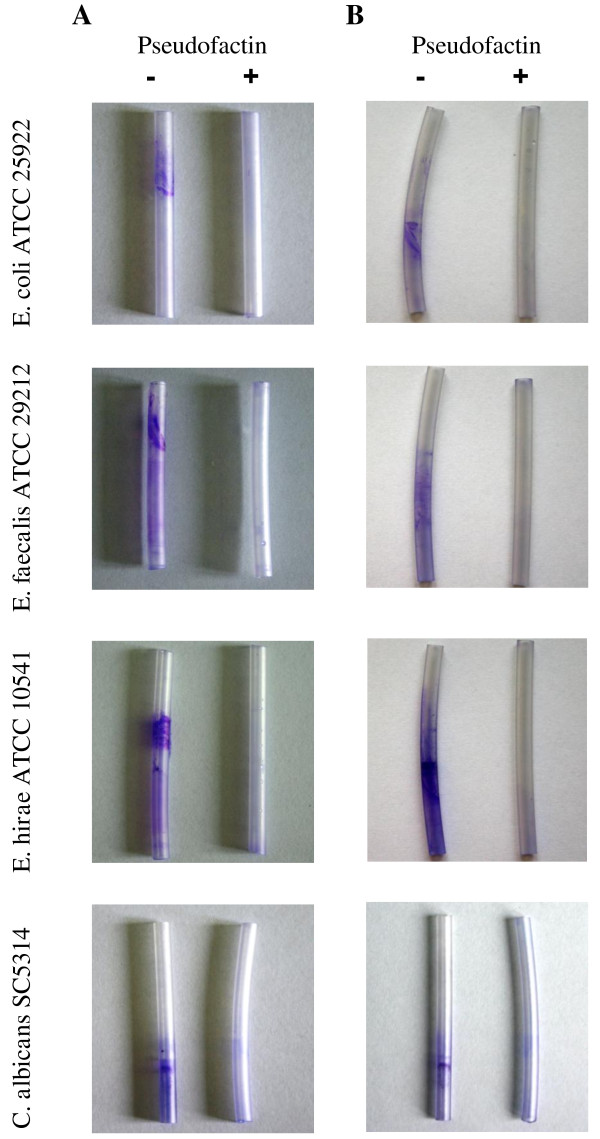
**Pseudofactin II inhibits biofilm formation on silicone urethral catheters**. The organisms were grown overnight at 37°C in a test-tube with sterile urethral catheters containing medium (A) with and without 0.25 mg/ml pseudofactin II and (B) where the urethral catheters was pre-incubated with biosurfactant at concentration 0.25 mg/ml as described in the text. Biofilms were visualized by staining with crystal violet.

The uropathogenic strains *E. coli, E. faecalis, E. hirae and C. albicans *were used in these tests. Ten microliter volumes of overnight cultures of *E. coli *ATCC 25922, *E. faecalis *ATCC 29212, *E. hirae *ATCC 10541 were added into 1000 μl of fresh LB medium, and the same volume of *C. albicans *SC5314 was added into 1000 μl of fresh RPMI-1640 medium. To the medium was added 1000 μl pseudofactin II (final concentration 0.25 mg/ml) solution in LB medium (for bacterial) and RPMI-1640 medium for *C. albicans *and 4 cm long segments of sterile silicone urethral catheters (Unomedical, Denmark). The catheters were incubated at 37°C overnight. The cultures were removed and the catheters were washed with distilled water. After washing, 3000 μl of crystal violet (0.1%) was added to the catheters for 20 min. The stained biofilms were rinsed three times with distilled water and allowed to dry at room temperature for 15 min before examination. In a parallel experiment the catheters were pretreated with pseudofactin II by being placed in a tube with 2000 μl of 0.25 mg/ml pseudofactin II dissolved in PBS, incubated for 2 h at 37°C and subsequently washed twice with PBS. Then the experiment was carried out as in the case of adding pseudofactin II into the growth medium. Assays were carried out two times. Representative images are presented on Figure 2. This experiment was carried out under dynamic conditions using a peristaltic pump, where the flow of culture with or without pseudofactin II trough urethral catheters was 50 ml/h.

## Results and discussion

### Antimicrobial activity of pseudofactin II

Lipopeptides have typical amphiphilic structure of a surfactant, where the hydrophobic moiety is a hydroxyl or α-alkyl-β-hydroxy fatty acid (e.g. 3-OH-C_14_, 3-OH-C_15 _and 3-OH-C_10 _fatty acids) and the hydrophilic moiety is a short chain or a cyclic peptide [22,23]. Instead, the hydrophobic moiety of pseudofactin II contains palmitic acid, which is a saturated fatty acid having no hydroxyl group. Rhodofactin, another lipopeptide with palmitic acid, has been described by Peng et al. [24]; however, contrary to pseudofactin II it has a short peptide chain which does not form lactone ring. Its antimicrobial activity has not yet been described.

The antimicrobial activity of pseudofactin II isolated from *P. fluorescens *BD5 was evaluated at concentrations from 0.035 to 0.5 mg/ml (Table 1). At 0.5 mg/ml the agent caused a total growth inhibition of *S. epidermidis *KCTC 1917 and considerable growth inhibition of *P. mirabilis *ATCC 21100 (37%), *E. coli *ATCC 10536 and *E. coli *17-2 (32%), *E. hirae *ATCC 10541 (28%).

**Table 1:** Growth inhibition obtained with the pseudofactin II isolated from *P.fluorescens *BD5 at different concentrations (mg/ml). Values ± confidence interval, n = 9

Microorganism	Growth inhibition (%)					
	**Pseudofactin II concentration (mg/ml)**					
	
	**0.500**	**0.250**	**0.200**	**0.150**	**0.075**	**0.035**

*Escherichia coli *ATCC 25922	8 ± 0.26	7 ± 0.52	6 ± 0.26	5 ± 0.39	3 ± 0.20	0 ± 0.26

*Escherichia coli *ATCC 10536	32 ± 0.26	28 ± 0.26	27 ± 0.39	18 ± 0.46	2 ± 0.26	2 ± 0.39

*Escherichia coli *17-2	32 ± 0.46	29 ± 0.33	24 ± 0.20	19 ± 0.20	14 ± 0.20	6 ± 0.20

*Enterococcus faecalis *ATCC 29212	18 ± 0.07	13 ± 0.07	11 ± 0.07	5 ± 0.13	5 ± 0.13	2 ± 0.07

*Enterococcus faecalis *JA/3	18 ± 0.07	15 ± 0.07	8 ± 0.07	4 ± 0.07	3 ± 0.07	0 ± 0.07

*Enterococcus hirae *ATCC 10541	28 ± 0.26	25 ± 0.33	22 ± 0.13	21 ± 0.46	10 ± 0.13	5 ± 0.52

*Staphylococcus epidermidis *KCTC 1917	100 ± 0.07	49 ± 0.07	44 ± 0.39	42 ± 0.20	16 ± 0.33	4 ± 0.26

*Proteus mirabilis *ATCC 21100	37 ± 0.33	36 ± 0.20	20 ± 0.20	17 ± 0.39	13 ± 0.13	0 ± 0.39

*Candida albicans *ATCC 20231	18 ± 0.26	17 ± 0.39	15 ± 0.46	15 ± 0.13	14 ± 0.26	11 ± 0.20

*Candida albicans *SC5314	9 ± 0.07	7 ± 0.07	5 ± 0.20	4 ± 0.213	1 ± 0.07	0 ± 0.07

In contrast to surfactin or iturin, produced by *B. subtilis *[25,26], lichenysin from *Bacillus licheniformis *[27] or polymyxin B and E from *Bacillus polymyxa *[28], pseudofactin II showed much weaker dose dependent antimicrobial activity against most strains tested in this work (Table 1). Only for two *Vibrio *strains pseudofactin II completely inhibited the growth in the lowest tested concentration, thus they were not used in further experiments (data not shown). This may be due to its unique chemical structure different from any currently known lipopeptides, which features a hydrophobic alkyl chain without a hydroxyl moiety attached to cyclic peptide.

### Antiadhesive and cell-dislodging activity of pseudofactin II on polystyrene surfaces

Adhesion of pathogenic microorganisms to solid surfaces or to infection sites has been found to be inhibited by biosurfactants capable of modifying the physico-chemical properties of the surface to reduce adhesion and biofilm formation on a given biomaterial.

Pseudofactin II was found to possess antiadhesive activity against all tested microorganisms. The pretreatment of polystyrene surfaces with pseudofactin II significantly decreased the adhesion of all bacteria and yeast, and this antiadhesive effect was concentration-dependent (Table 2). The highest reduction of adhesion (80-99%) was observed for *C. albicans *SC 5314, *C. albicans *ATCC 20231, *P. mirabilis *ATCC 21100 and *E. coli *ATCC 10536. The dislodging effect of pseudofactin II on preformed biofilms on untreated surfaces was lower than the preventive effect of pretreatment and was in the range of 26-70% for 0.5 mg/ml pseudofactin II (Table 3).

**Table 2:** Microbial adhesion inhibition in the microtiter plate by purified pseudofactin II.

Microorganism	Microbial adhesion inhibition (%)							
	**Pseudofactin II concentration (mg/ml)**							**Control (PBS)**
	
	**0.500**	**0.35**	**0.250**	**0.200**	**0.150**	**0.075**	**0.035**	**0**

*Escherichia coli *ATCC 25922	66 ± 0.13	65 ± 0.13	65 ± 0.07	64 ± 0.07	62 ± 0.07	58 ± 0.07	55 ± 0.20	0

*Escherichia coli *ATCC 10536	80 ± 0.13	80 ± 0.13	80 ± 0.13	77 ± 0.13	72 ± 0.13	65 ± 0.07	39 ± 0.13	0

*Escherichia coli *17-2	72 ± 0.33	71 ± 0.13	71 ± 0.13	69 ± 0.13	68 ± 0.07	64 ± 0.07	64 ± 0.13	0

*Enterococcus faecalis *ATCC 29212	70 ± 0.20	68 ± 0.20	68 ± 0.13	57 ± 0.13	55 ± 0.07	54 ± 0.07	42 ± 0.13	0

*Enterococcus faecalis *JA/3	36 ± 0.13	36 ± 0.13	34 ± 0.13	31 ± 0.13	22 ± 0.13	18 ± 0.20	15 ± 0.07	0

*Enterococcus hirae *ATCC 10541	71 ± 0.07	71 ± 0.20	71 ± 0.20	67 ± 0.20	66 ± 0.13	61 ± 0.07	58 ± 0.13	0

*Staphylococcus epidermidis *KCTC 1917	55 ± 0.13	45 ± 0.07	45 ± 0.07	33 ± 0.13	32 ± 0.07	31 ± 0.13	29 ± 0.13	0

*Proteus mirabilis *ATCC 21100	90 ± 0.20	90 ± 0.33	90 ± 0.33	89 ± 0.13	87 ± 0.07	85 ± 0.20	84 ± 0.20	0

*Candida albicans *ATCC 20231	92 ± 0.07	89 ± 0.07	81 ± 0.07	71 ± 0.13	68 ± 0.07	47 ± 0.20	45 ± 0.20	0

*Candida albicans *SC5314	99 ± 0.07	98 ± 0.07	98 ± 0.07	97 ± 0.07	96 ± 0.07	88 ± 0.07	87 ± 0.07	0

PBS was used as control and set at 0% as no microbial inhibition occurs. Values ± confidence interval, n = 9

**Table 3:** Activity of cell dislodging in the microtiter plate by pseudofactin II.

Microorganism	Microbial adhesion dislodging (%)							
	**Pseudofactin II concentration (mg/ml)**							**Control (PBS)**
	
	**0.500**	**0.35**	**0.250**	**0.200**	**0.150**	**0.075**	**0.035**	**0**

*Escherichia coli *ATCC 25922	66 ± 0.07	62 ± 0.07	62 ± 0.13	55 ± 0.07	42 ± 0.20	7 ± 0.07	4 ± 0.07	0

*Escherichia coli *ATCC 10536	64 ± 0.07	62 ± 0.13	61 ± 0.13	58 ± 0.07	50 ± 0.07	41 ± 0.07	38 ± 0.13	0

*Escherichia coli *17-2	70 ± 0.13	65 ± 0.13	59 ± 0.13	51 ± 0.07	46 ± 0.13	27 ± 0.07	18 ± 0.07	0

*Enterococcus faecalis *ATCC 29212	48 ± 0.07	42 ± 0.13	35 ± 0.13	33 ± 0.13	23 ± 0.13	20 ± 0.13	10 ± 0.07	0

*Enterococcus faecalis *JA/3	26 ± 0.26	23 ± 0.26	16 ± 0.26	15 ± 0.13	10 ± 0.07	6 ± 0.07	4 ± 0.20	0

*Enterococcus hirae *ATCC 10541	45 ± 0.13	43 ± 0.13	41 ± 0.33	35 ± 0.20	32 ± 0.20	31 ± 0.07	25 ± 0.13	0

*Staphylococcus epidermidis *KCTC 1917	43 ± 0.07	39 ± 0.26	37 ± 0.07	36 ± 0.07	23 ± 0.13	20 ± 0.13	17 ± 0.26	0

*Proteus mirabilis *ATCC 21100	45 ± 0.26	42 ± 0.26	40 ± 0.13	34 ± 0.13	28 ± 0.07	24 ± 0.07	21 ± 0.07	0

*Candida albicans *ATCC 20231	29 ± 0.26	22 ± 0.07	21 ± 0.07	16 ± 0.07	11 ± 0.07	6 ± 0.07	3 ± 0.07	0

*Candida albicans *SC5314	39 ± 0.07	31 ± 0.07	24 ± 0.13	20 ± 0.13	17 ± 0.07	7 ± 0.13	6 ± 0.13	0

Negative controls (PBS) were set at 0%. Values ± confidence interval, n = 9

The adhesion of pathogenic bacteria to polystyrene surfaces was inhibited by two lipopeptide biosurfactants produced by *B. subtilis *and *B. licheniformis *[9], and adhesion of *Listeria monocytogenes *to polystyrene microplates was reduced by 84% on pretreating the surface with surfactin (1 mg/ml), and by 82% when it was treated with purified rhamnolipid (7.5 mg/ml) [29]. Gudina et al. [30] characterized the anti-adhesive activity of biosurfactants against several microorganisms including Gram-positive and Gram-negative bacteria. This biosurfactant at concentration 25 mg/ml showed high anti-adhesive activity against *Staphylococcus aureus *(72.0%), *S. epidermidis *(62.1%), *Streptococcus agalactiae *(60.0%) and low anti-adhesive activity against *P. aeruginosa *(16.5%) and *E. coli *(11.5%).

Coating with pseudofactin II was effective above critical micelle concentration (0.072 mg/ml) [19]. Our results suggest that when the surface is covered by pseudofactin II micelles attached to polystyrene by van der Waals forces, the adhesion is inhibited more strongly than it is with monomers.

### Pseudofactin II reduces biofilm formation on polystyrene, glass and silicone

Biofilms are defined as microorganisms attached to a diverse range of biotic and abiotic surfaces and proliferating on them. The human body and medical devices or implants including: urinary catheters, voice prostheses, orthopedic implants, ocular prostheses and contact lenses are exposed to adhesion and biofilm formation by many opportunistic microorganisms. Thus we have tested the influence of pseudofactin II on biofilm formation on different materials.

The activity of pseudofactin II against biofilm formation was visualized by confocal laser scanning microscopy (Figure 1). The biofilm growth of *E. coli, E. faecalis, E. hirae *and *C. albicans *on polystyrene, glass and silicone from urethral catheters is shown in Figures 1A-D, 1I-L and 1R-U, respectively. The biosurfactant inhibited biofilm formation at the concentration 0.25 mg/ml on polystyrene, glass and silicone surfaces (Figures 1E-H, 1M-P and 1W-Z). *E. faecalis *ATCC 29212 adhesion to all tested surfaces is less intensive than others strains (Figures 1B, J, S). In fact, the adhesion of this strain to 96 wells plate was between 2 to 4-fold weaker than others tested bacterial strains (data not shown). This effect may be due to small amount of adhesion proteins on *E. faecalis *ATCC 29212 strain. Pseudofactin in the tested concentration inhibited mixed biofilm formation (data not shown), similarly as for the single strain (Figure 1). In our experiments all the tested Gram-negative and Gram-positive bacteria showed decrease of adhesion. The results of the present study indicate that pseudofactin II have potential to be used for efficient removal and inhibition of biofilms for pathogenic microorganisms.

Rivardo et al. [9] demonstrated that biosurfactants obtained from *Bacillus *spp. were able to inhibit biofilm formation for two pathogenic strains *E. coli *at 97% and *S. aureus *at 90%, respectively. Irie et al. [31] demonstrated that rhamnolipids produced by *P. aeruginosa *were able to disperse biofilm for *Bordetella bronchiseptica*.

### Pseudofactin II prevents biofilm formation in urethral catheters

To test biofilm formation on medical device, silicone urethral catheters, 4 cm segments of the catheters were incubated with *E. coli *ATCC 25922, *E. faecalis *ATCC 29212, *E. hirae *ATCC 10541 and *C. albicans *SC 5314. *E. coli, E. faecalis *and *E. hirae *formed biofilms mainly at the air-liquid interface, while the biofilm formed by *C. albicans *was dispersed along the whole growth surface (Figure 2). Even though the pseudofactin II present in the growth medium (Figure 2A), was at the concentration of 0.25 mg/ml which did not significantly affect the growth of the tested microbial cultures, biofilm formation was nearly completely prevented. The pretreatment of silicone urethral catheters with pseudofactin II prior to inoculation with medium was just as effective as including the biosurfactant in the growth medium (Figure 2B). We observed the similar effect in dynamic conditions for urethral catheters using a flow of 50 ml/h (data not shown).

Earlier reports noted an inhibition of biofilms formed by several microorganisms, e.g. *Salmonella typhimurium, S. enterica, E. coli *and *P. mirabilis *on vinyl urethral catheters by a surfactin produced by *B. subtilis *[32].

Our results show that pseudofactin II is promising compound for inhibition and disruption of biofilms and has potential applications in medicine.

## Conclusions

The biosurfactant pseudofactin II, produced by *P. fluorescens *BD5 strain and purified by HPLC, showed antiadhesive activity against several pathogenic microorganisms, such as *E. coli, E. faecalis, E. hirae, S. epidermidis, P. mirabilis *and *C. albicans*, which are potential biofilm formers on catheters, implants and internal prostheses. Up to 99% prevention of *C. albicans *SC 5314 adhesion could be achieved by 0.5 mg/ml pseudofactin II. Confocal laser scanning microscopy confirmed the action of pseudofactin II as an inhibitor of biofilm formation. In addition, pseudofactin II dispersed preformed biofilms. Due to its surface tension properties and lack of hemolytic activity (data not shown), pseudofactin II can be used as a surface coating agent against microbial colonization of different surfaces, e.g. implants or urethral catheters.

## Authors' contributions

TJ carried out experiments, ML participated in the design of the study, data analysis, coordination and helped to draft the manuscript, AK conceived the experiments and draft the manuscript. All authors read and approved the final manuscript.
